# Obesity and inflammatory markers in severe sepsis

**DOI:** 10.1186/cc11964

**Published:** 2013-03-19

**Authors:** P Simon, D Thomas-Rüddel, T Nemes, K Reinhart, F Bloos, UX Kaisers

**Affiliations:** 1 University Hospital of Leipzig, Germany; 2 Center for Sepsis Control and Care, Jena, Germany

## Introduction

Chronic inflammation has recently been recognized as an important factor in the pathophysiology of obesity and associated morbidities [1]. In this clinical study we aimed at identifying possible effects of obesity on inflammatory markers in severe sepsis.

## Methods

With institutional ethical committee approval, 243 consecutive patients treated for severe sepsis or septic shock in the ICUs of two university hospitals over a period of 5 months were studied. Six patients were excluded due to cachexia, syndromal disorders or missing clinical data. Diagnosis of sepsis was made according to SCCM criteria. Serum levels of C-reactive protein (CRP, mg/l) and procalcitonin (PCT, ng/ml) on day 1 of sepsis were compared among five body mass index (BMI) strata according to WHO definitions. Two groups (BMI <30, normal weight, and BMI ≥30, obesity) were formed for further analysis, and PCT was logarithmically transformed (LogPCT), resulting in normal distribution. Statistical analysis was performed using a *t *test.

## Results

**Figure 1:**
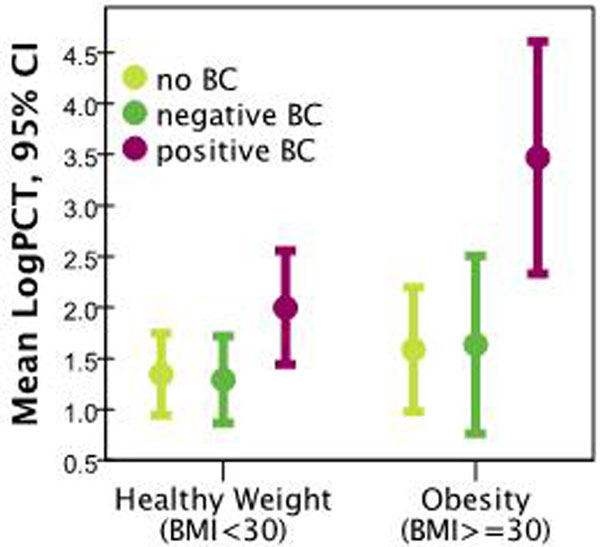


Patients with BMI ≥30 had higher values of PCT and CRP (Table 1). The difference in LogPCT was of borderline significance (*P *= 0.052). However, patients with positive blood cultures had significantly higher LogPCT values (*P *= 0.017) (Figure 1). Difference in CRP was not significant (*P *= 0.09). The trends over all five BMI strata (Table 1) were not significant.

**Table 1:** PCT and CRP as median (IQR)

BMI	*n*	PCT (ng/ml)	CRP (mg/l)
18.5 to 24.9	196	4.8 (11.9)	153 (189)
≥30.0	47	7.3 (24.6)	228 (254)
18.5 to 24.9	101	4.9 (11.7)	147 (175)
25 to 29.9	95	4.4 (12.0)	157 (218)
30 to 34.9	32	6.9 (22.6)	212 (118)
35 to 39.9	7	7.4 (38.3)	241 (264)
≥40.0	8	11.4 (32)	278 (272)

## Conclusion

Obesity with BMI ≥30 seems to be associated with an increase in inflammatory markers in patients with severe sepsis, particularly in bacteraemia. The role of adipose tissue in severe sepsis should therefore be studied in more detail.
